# Peritoneal flap for lymphocele prophylaxis following robotic-assisted laparoscopic radical prostatectomy with pelvic lymph node dissection: study protocol and trial update for the randomized controlled PELYCAN study

**DOI:** 10.1186/s13063-021-05168-x

**Published:** 2021-03-29

**Authors:** M. Neuberger, K. F. Kowalewski, V. Simon, F. Wessels, F. Siegel, T. S. Worst, N. Westhoff, J. von Hardenberg, M. Kriegmair, M. S. Michel, P. Honeck, P. Nuhn

**Affiliations:** 1 Department of Urology and Urologic Surgery, University Medical Center Mannheim, Medical Faculty Mannheim, University of Heidelberg, Theodor-Kutzer-Ufer 1–3, 68167 Mannheim, Germany; 2 Heinrich Lanz Centre for Digital Health, University Medical Center Mannheim, Medical Faculty Mannheim, University of Heidelberg, Mannheim, Germany

**Keywords:** Lymphocele, Robotic surgery, Prostate cancer, Prostatectomy, Randomized controlled trial, Evidence-based medicine, Urology

## Abstract

**Background:**

Data from interventional studies suggest that a peritoneal flap after pelvic lymph node dissection (LND) during laparoscopic, robotic-assisted radical prostatectomy (RARP) may reduce the rate of symptomatic lymphoceles in transperitoneal approach. However, most of these studies are not conducted in a randomized controlled fashion, thus limiting their scientific value. A recent prospective, randomized, controlled trial (RCT) did not show superiority of a peritoneal flap while further trials are lacking. Therefore, the aim of the presented RCT will be to show that creating a peritoneal flap decreases the rate of symptomatic lymphoceles compared to the current standard procedure without creation of a flap.

**Methods/design:**

PELYCAN is a parallel-group, patient- and assessor-blinded, phase III, adaptive randomized controlled superiority trial. Men with histologically confirmed prostate cancer who undergo transperitoneal RARP with pelvic LND will be randomly assigned in a 1:1 ratio to two groups—either with creating a peritoneal flap (PELYCAN) or without creating a peritoneal flap (control). Sample size calculation yielded a sample size of 300 with a planned interim analysis after 120 patients, which will be performed by an independent statistician. This provides a possibility for early stopping or sample size recalculation.

Patients will be stratified for contributing factors for the development of postoperative lymphoceles. The primary outcome measure will be the rate of symptomatic lymphoceles in both groups within 6 months postoperatively. Patients and assessors will be blinded for the intervention until the end of the follow-up period of 6 months. The surgeon will be informed about the randomization result after performance of vesicourethral anastomosis. Secondary outcome measures include asymptomatic lymphoceles at the time of discharge and within 6 months of follow-up, postoperative complications, mortality, re-admission rate, and quality of life assessed by the EORTC QLQ-C30 questionnaire.

**Discussion:**

The PELYCAN study is designed to assess whether the application of a peritoneal flap during RARP reduces the rate of symptomatic lymphoceles, as compared with the standard operation technique. In case of superiority of the intervention, this peritoneal flap may be suggested as a new standard of care.

**Trial registration:**

German Clinical Trials Register DRKS00016794. Registered on 14 May 2019.

**Supplementary Information:**

The online version contains supplementary material available at 10.1186/s13063-021-05168-x.

## Introduction and background

Pelvic lymphoceles present a common complication after RARP with lymph node dissection (LND) in approximately 10–18% of the cases [1–3]. Most lymphoceles are asymptomatic; nevertheless, in 1 to 15% of lymphoceles, symptoms occur. Obstruction of blood flow in the iliac vessels may cause thrombosis subsequently leading to venous thromboembolism. Hence, lymphoceles should be treated if they are symptomatic or impair venous blood flow. The current standard is percutaneous drain placement (ultrasound or computed tomography guided). However, in some cases, high flow rates of lymphatic fluid last for weeks thus causing substantial morbidity for the patient with additional hospital admission and loss in quality of life [1, 4–6]. In these cases, peritoneal fenestration is recommended [7].

To prevent lymphocele formation, especially lymphatic vessels at the level of the femoral canal are closed. For this purpose, different techniques have been introduced, which involve ligature, clipping, or mono- or bipolar sealing. However, these techniques are discussed controversially. On the one side, bipolar or monopolar coagulation may provide poor sealing of lymphatic vessels [8]. On the other side, no differences could be shown in a prospective randomized trial comparing clipping versus bipolar coagulation of lymphatic vessels at the level of the femoral canal [9]. However, these results are debated controversially [10, 11]. In addition, different approaches to reduce the rate of lymphoceles exist [12–15]. These strategies include intraoperative application of fibrin glue [12] or FloSeal® (Baxter International Inc., IL, USA), a hemostatic matrix that results in reduction of symptomatic lymphoceles [13]. However, the data regarding the clinical significance of hemostatic patches remains unclear, since the use of TachoSil® (Takeda Pharmaceutical Company Limited, Osaka, Japan) could not prove significant superiority [14]. Another study, which was assessing the benefits of using the Da Vinci® Vessel Sealer, a special endoscopic instrument by Intuitive Surgical (Sunnyvale, CA, USA), could not show a difference in the rate of postoperative lymphoceles (results not published on MEDLINE, ClinicalTrials.gov Identifier: NCT02035475). However, Yasumizu et al. indicated that using a vessel sealing device (EnSeal®, Ethicon Inc., Cincinnati, USA) can prevent the development of large lymphoceles. A difference in symptomatic lymphoceles could not be demonstrated [15]. Recently, published studies suggest that peritoneal fenestration or a peritoneal flap (PF) could decrease the risk for both asymptomatic as well as symptomatic lymphoceles due to resorption of lymph fluid by the peritoneum [6, 16–19]. In theory, this is caused by incision and folding over of the peritoneum into the region below of the field of the lymphadenectomy on both sides. This allows lymph fluid drainage from the true pelvis into the abdomen. On the one hand, the perivesical fat tissue no longer covers on the wound bed of the lymphadenectomy and lymph fluid can easily drain along the flap into the abdomen, and on the other hand, escaping lymph fluid can be reabsorbed via the peritoneum. Due to the methodical limitations of these studies (retrospective character, lack of randomization, insufficient statistical power), the efficacy of creating a PF remains unclear. In addition, in a recent study Bründl et al. report that implementation of a PF stays without effect on both symptomatic and asymptomatic lymphoceles [20]. However, in this trial sample, size calculation was based on the results by Lebeis et al. which might have been overoptimistic since a reduction to 0% is unlikely. At the time of submission, there are two further studies registered addressing lymphocele reduction after RARP by using a PF in a prospective setting (NCT03567525, DRKS00015720).

In conclusion, available evidence is limited and further high-quality RCTs are urgently needed.

Risk factors associated with lymphocele formation after RARP were identified in order to prevent imbalance between treatment groups. The following possible factors could be identified. However, evidence of most factors is controversially debated. The following possible factors could be identified or are under scientific debate:

- While prophylactic treatment with low molecular weight heparin seems to be associated with a higher lymphocele incidence [21, 22], heparin dosage (prophylactic vs. bridging) seems not to affect lymphocele rates [23]. Controversially, other data suggest no association between the formation of lymphoceles and prophylactic heparin regimen [24].
- A higher lymph node count has been shown to be predictive of a higher incidence of symptomatic lymphoceles [3, 22, 25, 26]. Furthermore, extended LND has been associated with a longer hospital stay [3] and a higher risk of lymphorrhea [26]. On the other hand, there is data, which suggests no association between node count and lymphoceles [27, 28].
- Diabetes mellitus seems to be associated with lymphocele development in general [29, 30] and was showed to be significantly associated with a higher risk of developing a superinfected post-prostatectomy lymphocele [31].
- Patients with symptomatic lymphoceles after RARP could be associated with a significant lower body mass index (BMI) [25].
- The use of either metallic clips or electrocoagulation could not be associated with a difference in lymphocele incidence after RARP so far [9].
- Drain placement seems not to affect the incidence of symptomatic lymphoceles [32].
- An age of > 65 years has been identified as a significant predictive marker for the occurrence of symptomatic lymphoceles [26].

### Objective and hypothesis

The aim of the PELYCAN study is to provide reliable evidence of the effectiveness of creating a PF after transperitoneal RARP with LND. The null hypothesis that will be tested in a confirmatory analysis is that the rate of symptomatic lymphoceles will be the same in both treatment groups or better in the control group.

## Methods

### Design

The PELYCAN study is a parallel-group, patient- and assessor-blinded, phase III, adaptive randomized controlled superiority trial with a 1:1 allocation ratio. The primary endpoint is the rate of symptomatic lymphocele 6 months after surgery.

### Study setting

The study will be performed at a tertiary university care center (University Medical Center Mannheim, Heidelberg University) in Germany. The study starts single-centered. Additional centers might be initiated during the trial. The country of recruitment and thereby data collection will be Germany. The study will be conducted according to the SPIRIT (Standard protocol items: Recommendations for Interventional Trials) Initiative [33] and CONSORT [34] guidelines. This study protocol follows the recommendations of the SPIRIT checklist [33]. The checklist has been included as Additional file 1.

### Trial population

All adult patients with an indication for elective transperitoneal RARP with regional or extended pelvic LND due to biopsy confirmed PCa will be screened for eligibility.

### Eligibility criteria

Patients must provide written informed consent before study participation (see Additional file 2 for sample informed consent form).

#### Patient inclusion criteria

Patients must

- (1) be newly diagnosed with histologically confirmed and clinically localized PCa and have chosen for laparoscopic, robotic-assisted radical prostatectomy (RARP)
- (2) provide mental capacity to consent
- (3) be at least 18 years old
- (4) be able to read and speak German

#### Patient exclusion criteria

- (1) previous laparoscopic inguinal hernia repair with mesh implantation
- (2) previous pelvic radiotherapy or major pelvic surgery
- (3) poor German language skills
- (4) metastasized disease
- (5) previous history of head injury, dementia, or psychiatric illness

### Interventions

#### Intervention description

Both groups receive standard transperitoneal RARP with pelvic LND. Whereas one group is attributed to the intervention (group A = PELYCAN), the other group (group B = control) will be the control group.

##### Group A: Intervention = PELYCAN

Following, the standard operative technique is described. Small variations according to the surgeon’s preference, such as the order of the right- and left-sided lymphadenectomy or using clips during lymphadenectomy and their amount, are allowed but must be recorded in the operation protocol. Radical prostatectomy will be performed by experienced high-volume surgeons. All surgeons use the same steps for LND, RARP, and the same vesicourethral anastomosis technique. The da Vinci® Xi Surgical System is used to perform the procedures.

**Fig. 1:**
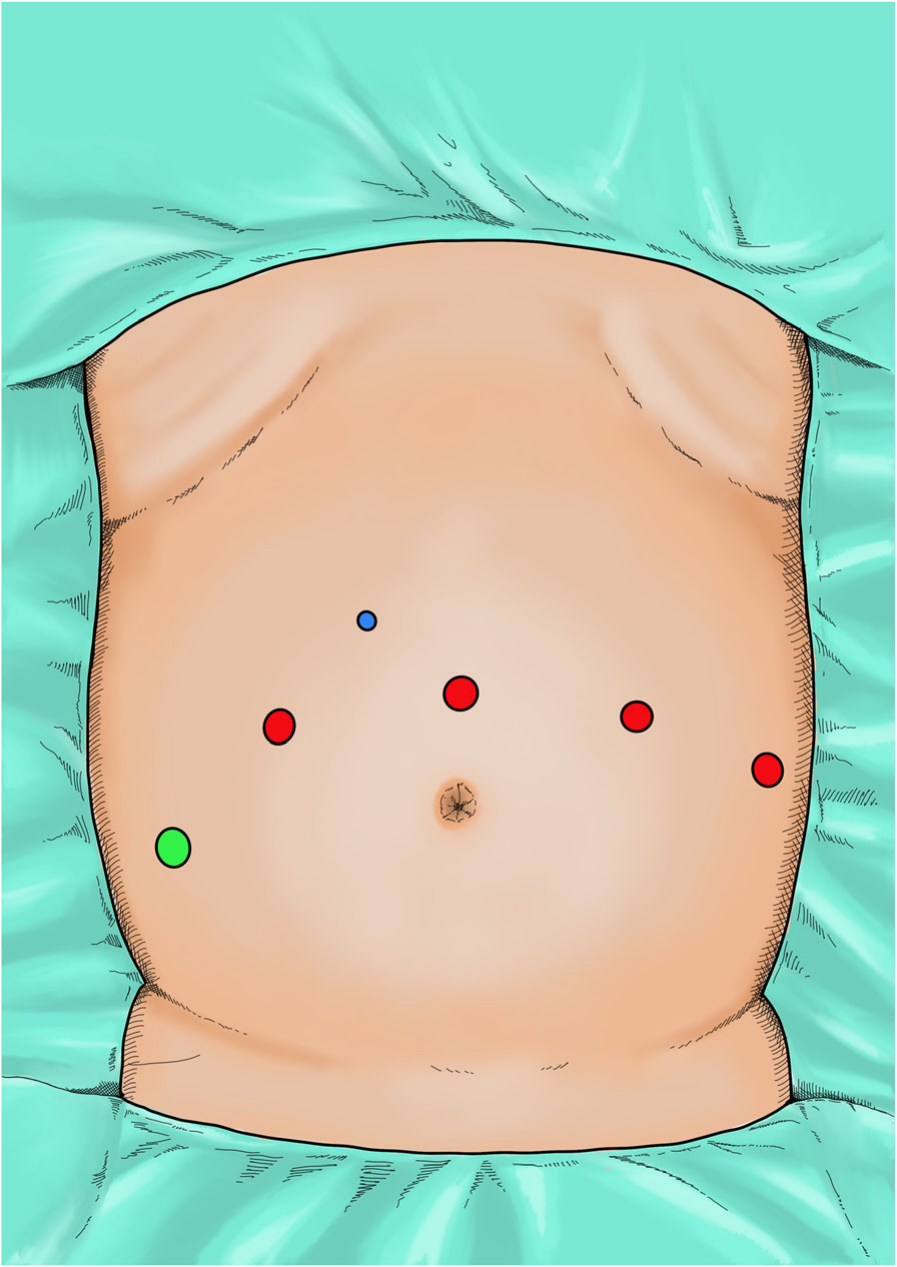
Trocar placement; blue = 5 mm assistant trocar, green = 11 mm assistant trocar, red = 8 mm da Vinci® trocars

**Fig. 2:**
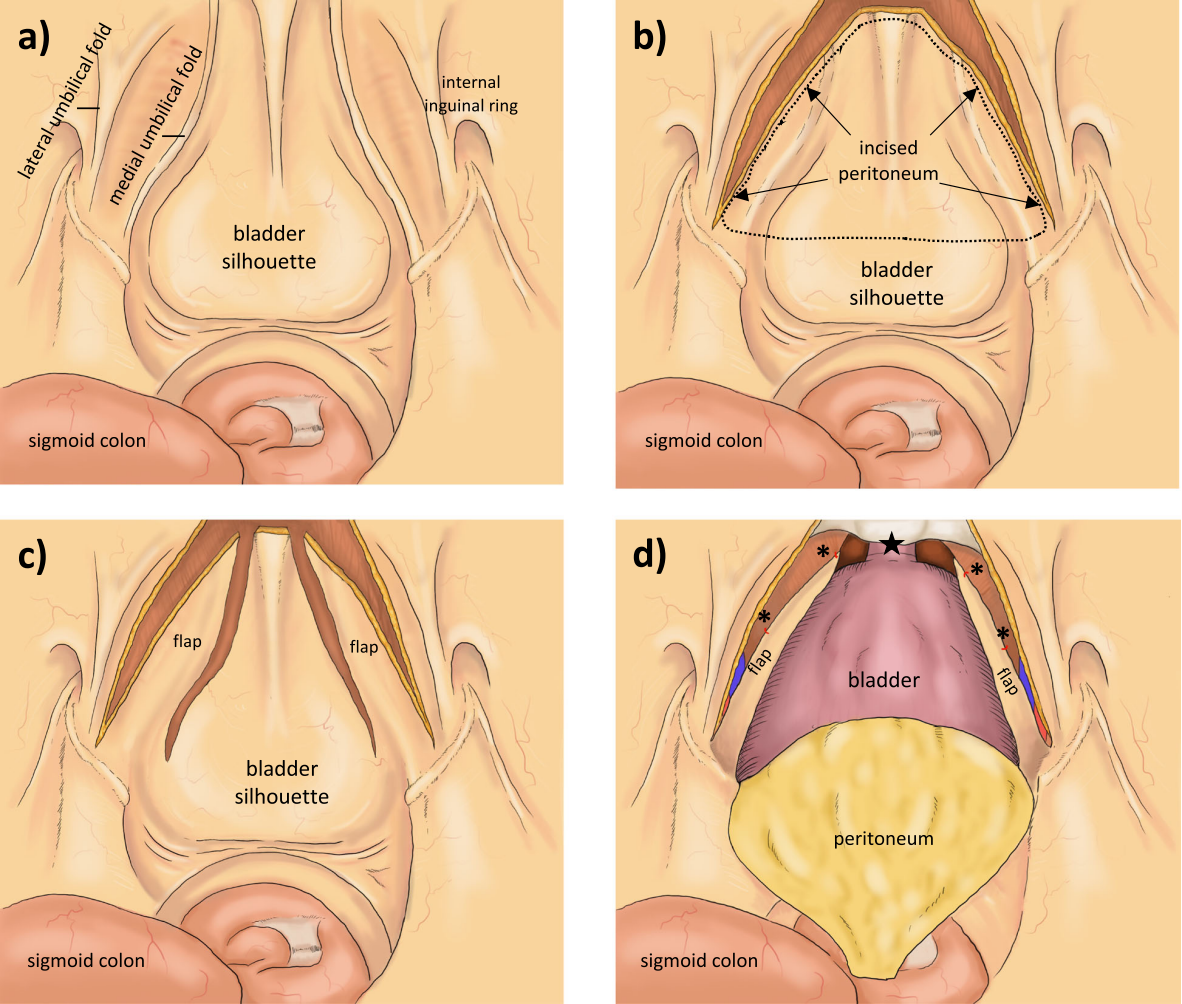
Intraoperative anatomy. **a** As seen from the cranial, intraperitoneal position of the camera. **b** After incision of the peritoneum along lateral umbilical fold. **c** After the PFs are created. **d** After fixation of the PFs. Asterisk indicates fixation to the pelvic floor using Vicryl 4/0 sutures; black star indicates vesicourethral anastomosis

Patients are under general anesthesia. The patient is placed in approximately 45° Trendelenburg position. Both arms are placed along the body. In total 6 trocars are placed in a modified, semicircular fashion (Fig. 1): 4 da Vinci® trocars (supraumbilical (8 mm trocar), left and right to the umbilicus (two 8 mm trocars), lower left hemiabdomen (8 mm trocar)) and 2 assistant trocars (right lower (11 mm trocar) and right upper (5 mm trocar) abdomen). Diagnostic laparoscopy is used to identify peritoneal adhesions prior to trocar placement. Peritoneum is incised next to the medial umbilical ligament. Next, the urachus is ligated, followed by preparation of the retropubic space down to the symphysis, which fully mobilizes the urinary bladder. Lateral umbilical ligament is incised to the spermatic duct on the left side. The procedure is repeated at the contralateral side (Fig. 2b). Afterwards, pelvic lymph node dissection is performed: The right internal inguinal ring is identified, followed by preparation of the lymph-node containing tissue in the obturator fossa between the external iliac vein and the obturator nerve. If assessed necessary by the surgeon, titanium clips (AESCULAP® Challenger® Ti-P, B. Braun, Melsungen, Germany) are used. In high-risk PCa extended LND (lateral margin: external iliac artery, cranial margin: bifurcation of the iliac artery) is performed. Lymph node dissection is performed at both sides. Lymph node tissue is placed in the ExBag® (ASID BONZ, Herrenberg, Germany) specimen pouch. After incision of the endopelvic fascia, the prostate is mobilized and suture ligation of the dorsal venous plexus is performed using one 4/0 Vicryl (Ethicon Inc., Cincinnati, USA) running suture. Bladder neck preparation is done in gentle technique with anterior incision.

The foley catheter can now be grabbed and moved ventrocranially. Now, the dorsal bladder neck is cut through. The anterior part of Denonvilliers’ fascia is severed, the spermatic duct and seminal vesicles identified, dissected, and held up/stretched. The anterior wall of the rectum can now be separated from the posterior part of the prostate either above or below the Denonvillier’s fascia depending on the nerve-sparing or non-nerve-sparing attempt. Depending on the preoperative agreement the neuro-vascular bundle is saved on both sides, one side or not saved in order to achieve higher oncologic safety. After apical separation, the urethra is incised and the foley catheter moved ventrally, which allows the separation of the posterior part of the urethrae. The prostate/specimen is placed in the ExBag® (Medtronic, Dublin, Ireland) specimen pouch. Bleeding is stopped using 4/0 Vicryl sutures and/or bipolar. After performing the dorsal Rocco stitch using a 3/0 V-loc™ barbed suture (Covidien, New Haven, USA), the vesicourethral anastomosis is sutured starting at 5 and 7 o’clock in lithotomy position and using a bidirectional 3/0 V-loc™ barbed suture (Covidien, New Haven, USA). Before the anastomosis is closed at 12 o’clock, a new foley catheter is inserted under visual control and the vesicourethral anastomosis is closed. Afterwards, the ventral Rocco stitch is performed. Next, the anastomosis is controlled for its integrity by filling the bladder with a minimum volume of 60 ml NaCl using the inserted catheter. Hereafter, the surgeon is informed about the result of patient randomization.

**Fig. 3:**
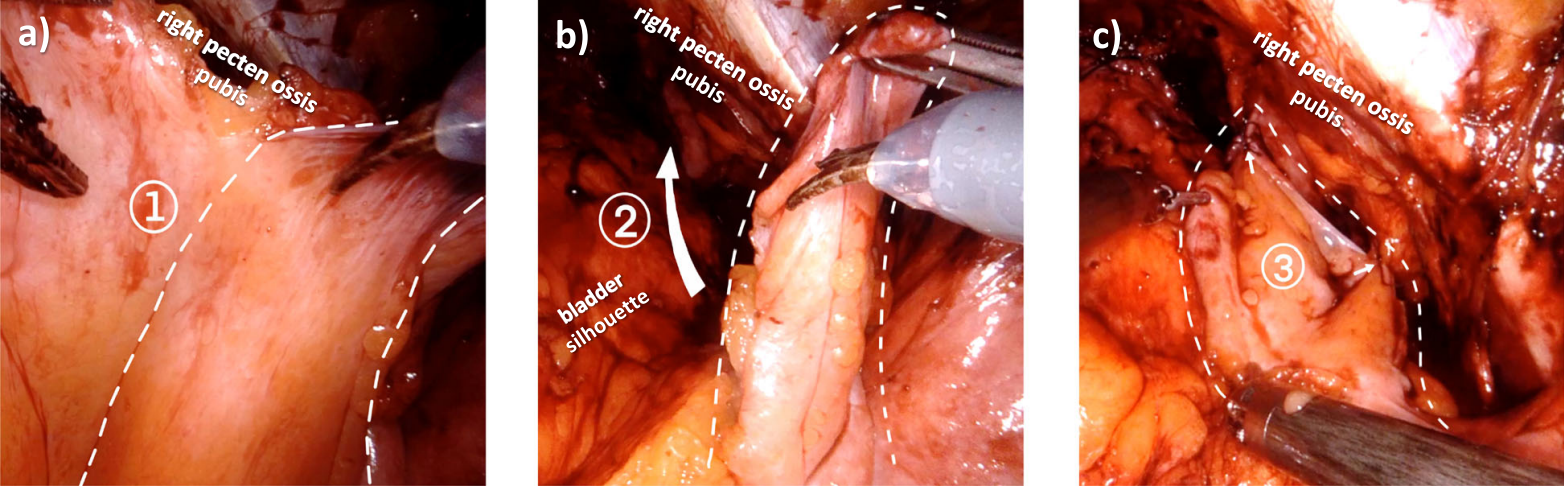
Intraoperative anatomy, as seen from the cranial. **a** The dashed line on the peritoneum indicates, where the incision for the PF is to be made. **b** The flap has been created; the big white arrow indicates where the PF is flapped over to. **c** The flap after its fixation, the two little white arrows indicate, where the PF has been fixed to the pelvic floor

The ventral peritoneum is incised laterally on both sides to create the PF (Fig. 2c). Those PF are fixated to the pelvic floor opposite to the region of the lymphadenectomy using 2 interrupted 4/0 Vicryl (Ethicon Inc., Cincinnati, USA) sutures (Fig. 2d). Figure 3 shows intraoperative pictures of the creation and fixation of the peritoneal flap on the right side.

As the last step, the operating field and the trocars’ entry points are inspected for bleeding or damages and the specimen is removed after expanding the entry point of the camera trocar. No wound catheters are placed, since the standardized procedure in RALP at our department does not include drain placement. This approach is supported by the result of a recent meta-analysis published in August 2020, which found a reduction in postoperative complications after radical prostatectomy when drainage was omitted (odds ratio (OR)[95% confidence interval (CI)]: 0.62[0.44;0.87], *p* = 0.006), while there were no differences for re-intervention (OR [CI]: 0.72[0.39;1.33], *p* = 0.300), lymphocele OR [CI]: 0.60[0.22;1.60], *p* = 0.310), hematoma (OR [CI]: 0.68[0.18;2.53], *p* = 0.570), or urinary retention (OR [CI]: 0.57[0.26;1.29], *p* = 0.180) between drainage and non-drainage, concluding that “the omission of drains can be recommended for standardized RP (...) cases [32]”. A more recent study from September 2020 comes to the same conclusion and states “pelvic drainage may be omitted after RALP without increasing postoperative complications or prolonging the hospital stay [35].”

##### Group B: Control

All the surgical steps taken are identical to the intervention group with the exception that no flaps are created after suturing the vesicourethral anastomosis. The surgeon is informed about the result of patient randomization at the same point in time. Instead of creating PFs, the situs remains as it is at the end of the operation with the previously incised peritoneum laying loose in its original position. The surgical technique in the control arm reflects the standard procedure. Small variations according to the surgeon’s preference, such as the order of the right- and left-sided lymphadenectomy or using clips during lymphadenectomy and their amount, are allowed but must be recorded in the operation protocol. No drainages will be placed.

#### Adherence

Since the PELYCAN study is an interventional study with a 6-month follow-up as well as a sonographic examination at the day of hospital discharge, we do not expect adherence problems. The sonographic examinations for lymphoceles reflect the department’s standard of care and are performed on all patients, who receive RARP with LND - independently of study participation.

#### Concomitant care

To us, there is no known concomitant care or intervention, which could possibly affect the outcome in terms of a cointervention bias. All patients receive the same pre-, peri- and postoperative care according to local standard operating procedures.

A difference exists in postoperative anticoagulation therapy depending on pre-existing medical conditions. Some patients are in need of therapeutic anticoagulatory whereas all others will receive a low molecular weight heparin for thrombosis prophylaxis for 4 weeks postoperatively as standard of care according to current guideline recommendations. Hence, we will stratify for therapeutic or prophylactic anticoagulatory therapy.

### Outcomes

#### Primary endpoint

- Difference between the two treatment arms in proportions of patients showing a symptomatic lymphocele within 6 months postoperatively. A symptomatic lymphocele is defined as sonographically detectable fluid accumulation in the area of the previously performed LND with the need of an interventional or operative therapy due to one of the following symptoms
  - Lower abdominal pain (after ruling out differential diagnosis)
  - Deep vein thrombosis/leg swelling/lymph drainage problems
  - Superinfection (fever/sepsis)

Data will be collected directly from the patients. These data will be complemented by the information gathered from the out-patient urologists, who treat the patients in an ambulant setting and perform their oncological follow-up.

#### Secondary outcome measures

For the secondary endpoint asymptomatic lymphocele at the time of discharge, ultrasonography will be performed by the assessors at the day of discharge. The assessors are blinded to the treatment group. Performing ultrasonography reflects the department’s standard of care. Asymptomatic lymphocele is defined as a sonographically detectable accumulation of fluid without symptoms. Furthermore, we will conduct a follow-up 6 months postoperatively. We will contact the patient directly. All patients gave informed written consent to be contacted. Lymphocele symptoms (pain, superinfection, deep vein thrombosis, or need for drainage insertion) will be assessed separately. Further secondary endpoints include:

- Rates of asymptomatic lymphoceles at the time of discharge
- The combined endpoint of asymptomatic lymphoceles at the time of discharge and symptomatic lymphoceles
- Quality of Life (QLQ-C30, EORTC)
- Rehospitalization rate within 6 months after surgical treatment
- Re-intervention rate
- Lymphedema and/or erysipelas
- Deep vein thrombosis
- Perioperative parameters (operating time, complication (Clavien-Dindo & Comprehensive Complication Index, blood loss)
- Length of hospital stay

#### Participant timeline

**Fig. 4:**
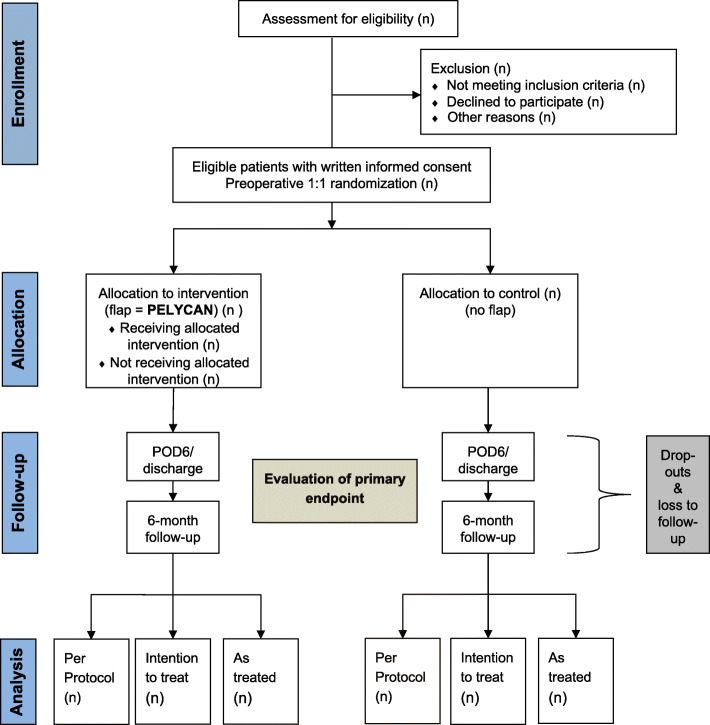
PELYCAN-trial flow diagram according to SPIRIT [33]

During the PELYCAN trial (Fig. 4), every patient, who will receive RARP will be handed patient, who will receive RARP will be handed out the patient education and informed consent materials, when he registers for his appointment with our secretary and trained research nurses. All participants receive the same information sheet and consent form. It is highlighted that the agreement to participate is voluntary. The patient is given enough time to study the participant education. All members of the admission team (nurses and admitting doctors) have been trained in detail about the study and its content. The admitting doctors are specialists only. All patients will be seen and screened by the admission doctor at their pre-admission appointment at our outpatient clinic. Their current medication is assessed and the QLQ-C30 (EORTC) is completed by the patients. All patients, who decide about receiving the operation on their own, are determined to have sufficient decisional capacity regarding trial participation. Participants not being able to speak or read German and patients with previous history of head injury, dementia, or psychiatric disease and patients lacking the decisional capacity for the operation itself are considered to lack the decisional capacity for study participation. If eligible, patients are invited to participate in the trial and informed consent is obtained. The patients’ questions will be addressed carefully.

Patients will receive ultrasonography examination at the day of discharge. The assessor will be the responsible ward doctor, who is blinded to group assignment. The main outcome interests are the occurrence of symptomatic lymphoceles. Patients will be assessed using questionnaires, which will be sent via email or post 6 months postoperatively. For data completion, we will also contact the patients’ outpatient urologists, who are responsible for the follow-up and medical/oncological aftercare. This increases the validity of our data. The questionnaires will be designed with questions which only can be answered dichotomously (“yes” or “no”). By that, we assure reproducible data. Additionally, patients will be sent the same quality of life (QoL) questionnaire they already have answered preoperatively (QLQ-C30, EORTC). Please refer to Table 1 for the detailed SPIRIT participant timeline.

**Table 1:** Participant timeline (adapted from the original table)

Timepoint	Study period						
	Staff members	−t1	0	t1.1	t1.2	t2	t3
Activity/assessment		Screening and consent	Allocation	Surgery	Peritoneal flap	Day of discharge	6-month follow-up
**Enroll**							
Eligibility screening	SC	x					
Informed consent	AD	x					
Allocation	SC		x				
**INT**							
Group A (PELYCAN)	OS			x	x		
Group B (control)	OS			x			
**Assessments**							
Pre-existing medical conditions	AD	x					
Medication	AD	x				x	
Abdominal ultrasound	AD	x				x	
Quality of life (QLQ-C30, EORTC)	AD, PU	x					x
Lymphocele (symptomatic/asymptomatic)	WD, PU					x	x
Lymphocele symptoms	WD, PU					x	x
Lymphedema/erysipelas	PU						x
Rehospitalization	PU						x

*Enroll* enrolment, *INT* intervention, *SC* study coordinator, *AD* admission doctor, *OS* operating surgeon, *WD* ward doctor, *PU* private urologist

#### Sample size

The sample size was calculated on the basis of the primary hypothesis. The incidence of symptomatic lymphoceles after RARP has been listed with 2–15% [6], 0–8% [17], and 9–51% [31, 36]. The incidence of post-prostatectomy *lymphoceles in general* has been described with an occurrence of 10–18%, [1], 30% [17], and up to 61% [37]. The previously conducted studies on PF for lymphocele prevention list the incidences for *symptomatic lymphoceles* in their control groups with 4.6% [16], 11.6 [6], 4.1% [17], 9,1% [20], and 9.7% [20], respectively. Stolzenburg et al. have been describing 8.3% *asymptomatic lymphoceles* in their matched-pair cohort; Bründl et al. described an incidence of asymptomatic lymphocele in 24.2% in the control group within the first 90 days postoperatively [16, 20].

Therefore, we assume a rate of symptomatic lymphoceles of 10% for the control group which can be reduced to 2% for the PELYCAN group. Using a power of 80% and a one-sided α (type 1 error) level of 0.025, we calculated a sample size of *n* = 141 per arm, in total *n* = 282. Assuming a drop-out rate of approximately 6%, we aim for a study population of 300 patients. However, due to uncertainty in the actual incidence of symptomatic lymphoceles and the effect of the intervention, sample size recalculation will be performed after an interim analysis in an adaptive study design after completion of the follow-up of the first 120 patients. According to the results of the sample size recalculation, the trial might be terminated for futility or efficacy by the PI or the sample size will be adapted [38, 39]. The maximum number of patients will be 1000.

#### Recruitment

Around 400 men undergo RARP at our department each year. In average, we estimate 30–35 patients per month. Six months after the start of recruitment, a total of 119 patients had agreed to participate. Therefore, we expect to enroll the initially planned 300 patients within 16–18 months. Recruitment is monitored by V.S. There are no financial or non-financial incentives provided to trial investigators or participants for enrollment.

#### Randomization

Eligible patients will be stratified for lymphocele risk factors and operating surgeon. Hereafter, randomization will be performed as block randomization with a 1:1 allocation to either using a PF (PELYCAN) or standard procedure without a PF (control). Block sizes will be kept confidential until study completion. Randomization and allocation will take place the day before surgery with a web-based computer algorithm (uroservy.de), which was specially developed for this study by the Heinrich-Lanz Center for Digital Health, Medical Faculty Mannheim, University of Heidelberg.

Randomization will be stratified for possible risk factors for lymphocele formation and the surgeon performing the procedure. The process of stratification is included in the “randomization process”: Patients are stratified for anticoagulation therapy (yes vs. no), extended lymphadenectomy (yes vs. no), diabetes mellitus (yes vs. no), and the surgeon performing the procedure. These categories have to be answered during the randomization process. Following this stratified proportionate sampling combined with randomization, we aim to ensure an equal allocation of participants to each experimental condition (intervention vs. non-intervention). That is done to control for the possible confounding variables regarding postoperative lymphocele formation (e.g., preventing that significantly more patients with extended LAD are randomized into the non-intervention group). Stratification based on intraoperative patient characteristics was considered impossible. To avoid over-stratification, we limited the number of strata. Aiming for a high number of patients, blocking within the strata will be performed. All patients included will be used for statistical analyses.

The randomization and allocation process will be performed by a person, who is completely uninvolved in the running of the trial, the study inclusion, the process of taking the patients’ consent, the operation or postoperative assessment of lymphoceles. This person cannot be influenced by people involved in the trial.

All randomized study participants will stay included in the trial. Follow-up information will be collected in order to prevent missing data.

Concealment is achieved by using the web-based computer algorithm mentioned above. Permuted-block randomization will be used for equal proportions regarding the provided treatment. Block sizes will be kept confidential until study completion, to ensure concealment. Only the surgical assistant is informed about the study inclusion and allocation the evening before the operation takes place. By blocking within the strata, allocation becomes even more unpredictable for the surgeon.

Whether or not the intervention has been performed is concealed by using a code, which is assigned to each patient individually. A list of patients and their matching codes is provided in a separate document. Allocation will not be released until the end of the 6-month follow-up, ensuring concealment. Enrollment will be performed by the admission doctor as mentioned above.

#### Blinding and blinding mechanism

To reduce performance bias, patients and assessors will be blinded to group allocation until 6 months after surgery [40]. Randomization will take place 1 day before surgery. The operating surgeon will be informed about group allocation after the vesicourethral anastomosis is finished. This late point of involving the surgeon guarantees a consistent procedure and thereby minimizes the surgeon’s bias.

By using a unique randomization ID for each patient, patients, outcome-assessors for in-patient treatment and examination of lymphoceles as well as the care providers/outcome-assessors for examination and follow-up after discharge will be blinded. Data collectors will be kept blinded as well until the data collection will be finished.

##### Emergency unblinding

To maintain the overall quality and legitimacy of the clinical trial, code breaks should occur only in exceptional circumstances when knowledge of the actual treatment is absolutely essential for further management of the patient. If unblinding is deemed to be necessary, the investigator should use the system for emergency unblinding through contacting the PI or the people responsible for study coordination. The investigator is encouraged to maintain blind as long as possible. The actual allocation must not be disclosed to the patient and/or other study personnel including other site personnel nor should there be any written or verbal disclosure of the code in any of the corresponding patient documents. The Investigator must report all code breaks (with reason) as they occur.

### Data collection

#### Trial procedures and evaluations

##### Primary outcome

The primary endpoint will be identified by directly asking the patient. Since a symptomatic lymphocele provides symptoms, we consider the validity and reliability of this approach as high. The same approach will be used for drainage insertion and deep vein thrombosis. Therefore, patients will be contacted and sent a questionnaire by email. If the primary endpoint was met or patients will not respond to the email, they will be contacted by another email, per letter or telephone.

To promote data quality the patients’ follow-up specialists will be contacted and asked for the occurrence of the primary endpoint. Contacting patients and their follow-up specialists will have been allowed in the written informed consent.

##### Secondary outcomes

Sonographic evaluation for lymphoceles is included in the discharge examinations. All results are noted in the discharge letter. Therefore, we have no doubt that assessing for “asymptomatic lymphocele at the day of discharge” will have high validity and reliability. The occurrence of “asymptomatic lymphocele,” “lymphedema,” “erysipelas,” and “rehospitalization” is included in the questionnaire mentioned above. Additionally, patients will receive the validated EORTC QLQ-C30 and the Lymphoedema Functioning, Disability and Health Questionnaire for Lower Limb Lymphoedema (Lymph-ICF-LL).

##### Training plans

All assessors were instructed about the necessary measures at discharge and how to document each study parameter.

#### Retention

By contacting the patients using different methods, we expect to have low rates of patients “lost to follow-up.” By asking the patients’ follow-up specialist, we create a “double measurement.” That improves retention and aims to decrease the number of patients “lost to follow-up.”

Since the intervention is an operative procedure at the beginning of the study period, non-adherence is no matter of discussion in our cohort. Once a patient is enrolled or randomized, the study site will make every reasonable effort to follow the patient for the entire study period.

##### Participant withdrawal

Participants may withdraw from the study for any reason at any time. The investigator also may withdraw participants from the study in order to protect their safety (e.g., if the planned PF is not possible due to anatomical reasons or an anesthesiologic need for finishing the procedure without artificial prolonging the operation time).

#### Data management

##### Data forms and data entry

In the PELYCAN trial, all data will be entered electronically in case report forms (eCRFs). This will be done at the Department of Urology at the University Medical Center Mannheim. Personal information about potential and enrolled participants will be collected, shared, and maintained with third-party only after pseudonymization in order to protect confidentiality. All demographic and baseline clinical data, as well as primary and secondary outcome measures, will be recorded in the eCRF. Original study forms (e.g., those who will be sent to the patients for follow-up either electronically or by post) will be entered and kept on file at the department. Data management will be done by V.S. and the study center of the department of urology and urologic surgery.

##### Data transmission and editing

To promote data integrity, a variety of mechanisms will be used. Referential data rules, valid values, range checks, and consistency checks against data already stored in the database will be supported. The option to choose a value from a list of valid codes or options will be available where applicable. Checks will be applied at the time of data entry into a specific field.

##### Storage, security, and back-up of data

Data will be stored in compliance with data protection regulations. Access to the study data will be restricted. All forms related to the study will be kept in locked cabinets. The electronic data forms will be password-protected. These passwords will be changed on a regular basis. All reports will be prepared such that no individual subject can be identified by using the patients “hospital-ID.” A complete back-up of the primary database will be performed twice monthly. These back-ups will be stored off-site. A complete back-up of the primary database will be retained indefinitely.

### Statistical methods

#### Outcomes

The intervention arm (PELYCAN) will be compared against the control for all primary analyses. The primary analysis will be based on the intention-to-treat (ITT) principle. In addition, per protocol (PP) and as-treated (AT) analyses will be performed as sensitivity analysis. We will use chi-squared test for binary outcomes and *t* test for continuous outcomes. Categorical data will be reported with absolute and relative frequencies. Continuous outcomes will be reported with mean/median as well as standard deviation and (interquartile)-range. For subgroup analysis, we will use regression methods. Multivariable analyses will be based on logistic regression for binary outcomes and linear regression for continuous. *P* values will be reported to four decimal places with *p* values less than 0.001 reported as *p* < 0.001. Up-to-date versions of SPSS (Chicago, IL) and R will be used to conduct analyses. For the primary outcome, the *p* value will be adjusted according to the adaptive design in order to avoid a type I error. All other analyses will be exploratory with 2-sided *p* values of alpha < 0.05 and a power of 80%. Statistical analysis will be performed by a statistician who is otherwise not involved in the conduct of the study.

#### Additional analyses

We plan to conduct subgroup analyses with strong biological rationale and possible interaction effect. The subgroup analysis will compare odds ratios for the use of titanium clips during lymphadenectomy versus lymphadenectomy without the use of clips.

A sensitivity analysis of the primary endpoint will adjust for the pre-randomization variables which might reasonably be expected to be predictive of outcomes.

#### Missing data

Since the intervention is an operative procedure at the beginning of the study period non-adherence is no matter of discussion in our cohort. Once a patient is enrolled or randomized, the study site will make every reasonable effort to follow the patient for the entire study period. If a patient is not willing to send back the information of the follow-up after 6 months, we will report reasons for withdrawal for each randomization group and compare the reasons qualitatively.

### Data monitoring

#### Formal committee

No data monitoring committee will be installed. However, in order to ensure patient safety, all perioperative complications will be assessed and analyzed.

#### Safety and harms

We do not expect any unexpected or unexplained harms or adverse events. In our study, adverse events will be defined as any unfavorable medical occurrence in a subject without regard to the possibility of a causal relationship. Adverse events will be collected from day of operation until the day of discharge from our department and at the 6-month follow-up after the subject has provided written informed consent an enrolled in the study.

Most adverse events that are seen in patients after RARP are anticipated (symptomatic lymphocele, deep vein thrombosis, drainage insertion, urinary tract infection, hematuria, lymphedema, and erysipelas). However, all adverse events will be recorded. Serious adverse events (SAE) between study enrollment and hospital discharge will be reported. A SAE for this study is any untoward medical occurrence that is believed by the investigators to be causally related to the intervention and results in any of the following: life-threatening condition (immediate risk of death), severe or permanent disability, or prolonged hospitalization. Investigators will determine relatedness of an event to intervention as well as whether the event is unexpected or unexplained given the subjects’ clinical course and previous medical conditions.

The investigators inform the principal investigator (PI) about harms. Depending on harms, the PI might inform the local ethics committee and terminate the study.

#### Auditing

There will not be any auditing. Since there is no sponsor and the investigators do not have a financial nor a non-financial incentive neither, the study will be performed independently.

## Ethics and dissemination

### Research ethics approval

Ethical approval has been obtained from the University of Heidelberg’s Ethics Committee II (Medical Faculty Mannheim 2019-1127 N (Additional file 3)) with respect to scientific content and compliance with applicable research and human subjects’ regulations. The study protocol, participant education, and the informed consent form have also been reviewed by that ethical committee. Written, informed consent to participate will be obtained from all participants.

### Protocol amendments

If any modifications to the protocol will be necessary, which may have an impact on the conduct or the potential benefit of the study or affect the patient safety, a formal amendment to the protocol will be required. These modifications include changes of the study objectives, study design, patient population, study procedures, or significant administrative aspects.

The amendment will be agreed upon by the PI and it has to be approved by the ethics committee prior to implementation. Any of these modifications will be described in the trial report to prevent bias and provide trial integrity.

### Ancillary studies

Every participant provides written consent for ancillary studies. This is included in the informed consent material. Therefore, and due to the fact that no biological specimens will be taken, no additional consent is needed.

### Confidentiality

All study-related information will be stored securely at the study site. All participant information will be collected in strictly pseudonymous form and be stored in a password-protected file. The follow-up document, if not obtained electronically, will be stored in a locked file cabinet in areas with limited access. All reports, data collection, process, and administrative forms will be identified by a coded ID only to maintain participant confidentiality. The data and the linking code will be stored in separate locations using encrypted digital files within password-protected folders and storage media. All records that contain names or other personal identifiers will be stored separately from study records. All local databases will be password-protected. Participants’ study information will not be released outside of the study without the written permission of the participant. The access to the data is limited to the minimum number of individuals necessary for quality control and analysis. There will not be any data transmission.

### Declaration of interests

All authors declare that they have neither financial nor other competing conflicts of interest.

### Access to data

The principal investigator will be given access to the full data sets. To ensure confidentiality, data dispersed to all other investigators and project team members will be blinded of any identifying participant information.

### Ancillary and post-trial care

Due to the operative-interventional character of the study access to the intervention (peritoneal flap) cannot be provided to the control group. Since we do not expect any complications, we do not consider post-trial or ancillary care necessary.

### Dissemination policy

#### Trial results

The results of the PELYCAN trial will be submitted to a peer-reviewed journal regardless of the study outcome. Every attempt will be made to reduce to an absolute minimum the interval between the completion of data collection and the release of the study results. We expect to take about 3–6 months to compile the final results paper for an appropriate journal.

#### Authorship

Authorship will be based on the ICMJE (International Committee of Medical Journal Editors) recommendations. Participants who do not fulfill the authorship criteria will be listed as “collaborator.”

### Trial status

The first patient was randomized on September 2, 2019. At the time of the protocol submission (August 16th, 2020), our center was actively recruiting patients for the trial and 140 of 300 patients had been randomized. Inclusion is according to schedule. This protocol version is 1.0 (October 17th, 2020).

### Sponsor and funder

The PELYCAN study is an investigator-initiated trial. It will not have any sponsors or funding sources except for partial financial support for open access publication costs by the Baden-Württemberg Ministry of Science, Research and the Arts and by Ruprecht-Karls-Universität Heidelberg.

Hence, sponsors or funding will have no role in the design or conduction of the PELYCAN study.

## Discussion

RARP is a surgical gold standard for localized PC patients. In most cases, it is performed in combination with LND. Symptomatic lymphoceles are a common complication after LND. Different approaches have been described to reduce lymphocele-development after RARP [12–15]. A recently published systematic review could not find evidence among these methods [41]. However, they stated that peritoneal reconfiguration could present a reasonable option and called for further data [41]. This has been suggested in several studies [6, 16–19]. The only RCT, which has been published recently, showed no difference in symptomatic lymphoceles regarding the creation of a PF [20]. However, that study was limited due to a low sample size [20].

In a thorough research including the German Clinical Trials Register and ClinicalTrials.gov, we could identify only two ongoing RCTs assessing peritoneal flaps in order to prevent or decrease lymphoceles after RARP with LND. One of these is a multicenter study (DRKS00015720), whereas the other is designed single center (NCT03567525). Both the studies’ primary endpoint is the prevalence of lymphoceles within 90 days postoperatively. While their primary endpoint includes both asymptomatic and asymptomatic lymphoceles, the primary endpoint of the PELYCAN study is symptomatic lymphoceles. Furthermore, lymphoceles which form > 90 days postoperatively will not be covered by these studies. Additionally, none of the mentioned RCTs has published study protocols.

The investigator-initiated PELYCAN study is an RCT to compare RARP and LND with creation of PF versus RARP and LND. It focuses on the difference of postoperative lymphoceles. The primary endpoint is symptomatic lymphoceles within 6 months. This endpoint is one strength of the PELYCAN study as it is highly objective and standardized.

The PELYCAN study consists of one intervention, which is the creation of a PF. The PF is only created in the intervention group and performed by bilateral incision of the peritoneum with subsequent fixation at the pelvic floor. For fixation 2 interrupted 4/0 Vicryl sutures are used. This is an easy, suitable and expeditious procedure. To our knowledge, this procedure does not produce any harms.

Blinding of patients in surgical trials usually is difficult. Given the fact that the intervention does not include the surgical access, patient blinding will be possible without limitations.

One point of discussion is that due to the nature of the intervention, a complete double-blinding in terms of blinding the surgeon is impossible. However, until the vesicourethral anastomosis is finished, the surgeon will remain blinded. This late point of unblinding guarantees a consistent procedure and thereby lowers the surgeons’ bias. Additionally, outcomes assessors will be blinded until 6 months postoperatively.

In conclusion, the PELYCAN study is a parallel-group, patient- and assessor-blinded, phase III, adaptive randomized controlled superiority trial investigating the impact of PFs on symptomatic lymphoceles following RARP with LND performed by experienced surgeons beyond the learning curve. The PELYCAN study has the potential to initiate a paradigm shift in order to avoid postoperative lymphoceles and further refine the technique of RARP.

## Supplementary Information

**Additional file 1.** Completed SPIRIT 2013 checklist.

**Additional file 2.** Model informed consent form.

**Additional file 3.** Ethical approval.

## Data Availability

The principal investigator and M.N. will be given access to the full data sets. To ensure confidentiality, data dispersed to all other investigators and project team members will be blinded of any identifying participant information.

## Abbreviations

AT: As treated
BMI: Body mass index
CONSORT: Consolidated Standards Of Reporting Trials
EORTC: European Organisation for Research and Treatment of Cancer
ITT: Intention to treat
LND: Lymph node dissection
PF: Peritoneal flap
PI: Principal investigator
PP: Per protocol
QoL: Quality of life
RARP: Robotic-assisted radical prostatectomy
RCT: Randomized, controlled trial
SAE: Serious adverse event
SPIRIT: Standard Protocol Items: Recommendations for Interventional Trials
