# Supplementary material for: Peritoneal flap for lymphocele prophylaxis following robotic-assisted laparoscopic radical prostatectomy with pelvic lymph node dissection: study protocol and trial update for the randomized controlled PELYCAN study
Source: Trials. 2021 Mar 29;22:236. doi: 10.1186/s13063-021-05168-x (PMC8008541; doi:10.1186/s13063-021-05168-x)
Supplement: Supplementary file 2 — Additional file 2. Model informed consent form. [file 13063_2021_5168_MOESM2_ESM.doc]

# Einwilligungserklärung (Version 1.1 vom 25.03.2019)

**Einwilligungserklärung**

**Peritonealflap (Bauchfell-Umschlag) zur Prophylaxe von Lymphozelen (Lymphflüssigkeitsansammlungen) nach laparoskopischer, robotisch-assistierter, radikaler Prostatektomie mit pelviner Lymphknotendissektion (Lymphknotenentfernung im Becken)**

Name des Patienten: ____________________________________________________________

E-Mail-Adresse des Patienten: ____________________________________________________

Prüfärztin/Prüfarzt: Frau/Herr Dr.med. ______________________________________________

hat mit mir heute ein ausführliches Aufklärungsgespräch über das Projekt geführt. Dabei wurden u.a. Studienziel und Studienlänge, studienbedingte Erfordernisse und mögliche Nebenwirkungen besprochen. Die Patienteninformation sowie ein Exemplar der Einwilligungserklärung habe ich erhalten, gelesen und verstanden. In diesem Zusammenhang bestehende Fragen wurden besprochen und beantwortet. Ich hatte ausreichend Zeit, mich freiwillig für oder gegen eine Teilnahme an dieser Studie zu entscheiden.

**Ich willige ein, als Patient an dieser klinischen Studie teilzunehmen.**

Mir ist bekannt, dass diese Studie in erster Linie der medizinischen Wissenserweiterung dient und gegebenenfalls auch keinen persönlichen Vorteil für mich bringen kann.

Ich bin darüber unterrichtet worden, dass meine Teilnahme vollkommen freiwillig erfolgen muss und ich meine Einwilligung zur Teilnahme an diesem Projekt jederzeit ohne Angabe von Gründen und ohne persönlichen Nachteil widerrufen kann. Auch der Arzt kann aufgrund seiner ärztlichen Erfahrung die Prüfung jederzeit beenden.

Ich bestätige meine Teilnahme an dieser Studie. Alle Daten werden streng vertraulich gehalten. Ich weiß, dass ich keine finanziellen Zuwendungen oder Patentrechte erhalte.

**Ich willige ein, dass ich oder mein behandelnder Hausarzt/ Facharzt evtl. zu einem späteren Zeitpunkt erneut kontaktiert werden *zur Gewinnung weiterer Informationen* und *zur Rückmeldung gesundheitsrelevanter Ergebnisse* (falls nicht gewünscht, jeweiligen Abschnitt bitte streichen**

**Datenschutz**

Mir ist bekannt, dass bei dieser Studie personenbezogene Daten verarbeitet werden sollen. Die Verarbeitung der Daten erfolgt nach gesetzlichen Bestimmungen und setzt gemäß Art. 6 Abs. 1 lit. a der Datenschutz-Grundverordnung folgende Einwilligungserklärung voraus:

Ich wurde darüber aufgeklärt und willige ein, dass meine in der Studie erhobenen Daten, insbesondere Angaben über meine Gesundheit, zu den in der Informationsschrift beschriebenen Zwecken pseudonymisiert in Papierform oder auf elektronischen Datenträger in der Klinik für Urologie der Universitätsklinik Mannheim aufgezeichnet und ausgewertet werden.

Ich willige ein, dass die entsprechend berechtigten Mitarbeiter im Rahmen des beschriebenen Projekts gegebenenfalls auch (zukünftig) Angaben über meine Gesundheit von mir erfragen oder aus meinen Krankenunterlagen entnehmen kann.

Eine eventuelle Weitergabe der Daten erfolgt nur in pseudonymisierter Form, nur im Rahmen einer wissenschaftlichen Veröffentlichung und nur innerhalb Deutschlands, Europas (EU) oder des Europäischen Wirtschaftsraumes. Dritte erhalten keinen Einblick in personenbezogene Unterlagen. Die Klarnamenliste verbleibt in der Klinik für Urologie, Mannheim.

Bei der Veröffentlichung von Ergebnissen der Studie wird mein Name ebenfalls nicht genannt. Die personenbezogenen Daten werden anonymisiert, sobald dies nach dem Forschungszweck möglich ist. Die Daten werden nach Studienabschluss 5 Jahre aufbewahrt

Mir ist bekannt, dass diese Einwilligung jederzeit schriftlich oder mündlich ohne Angabe von Gründen widerrufen werden kann, ohne dass mir dadurch Nachteile entstehen. Die Rechtmäßigkeit der bis zum Widerruf erfolgten Datenverarbeitung wird davon nicht berührt. In diesem Fall kann ich entscheiden, ob die von mir erhobenen Daten gelöscht werden sollen oder weiterhin für die Zwecke der Studie verwendet werden dürfen.

Ich habe eine Kopie der Patienteninformation und Einwilligungserklärung erhalten.

*Name der des Patienten Ort Datum Unterschrift des Patienten*

**Aufklärende Person**

Der Patient wurde von mir im Rahmen eines Gesprächs über das Ziel und den Ablauf der Studie sowie über die Risiken aufgeklärt. Ein Exemplar der Informationsschrift und der Einwilligungserklärung habe ich dem Patienten ausgehändigt.

*Name der/des aufklärenden Ärztin/Arztes Ort Datum Unterschrift der/des aufklärenden Ärztin/Arztes*
