# Supplementary material for: Peritoneal flap for lymphocele prophylaxis following robotic-assisted laparoscopic radical prostatectomy with pelvic lymph node dissection: study protocol and trial update for the randomized controlled PELYCAN study
Source: Trials. 2021 Mar 29;22:236. doi: 10.1186/s13063-021-05168-x (PMC8008541; doi:10.1186/s13063-021-05168-x)
Supplement: Supplementary file 3 — Additional file 3. Ethical approval. [file 13063_2021_5168_MOESM3_ESM.pdf]

UMM UNIVERSITÄTSMEDIZIN MANNHEIM

Medizinische Fakultät Mannheim
der Universität Heidelberg

Universitätsklinikum Mannheim

UMM Universitätsmedizin Mannheim
Med. Ethik-Kommission II, Theodor-Kutzer-Ufer 1-3, 68167 Mannheim

Herrn
Prof. Dr. med. Philipp Nuhn
Urologische Klinik
Theodor-Kutzer-Ufer 1-3

68167 Mannheim

Ethik-Kommission II der Universität Heidelberg
Medizinische Fakultät Mannheim

Vorsitzender: **Prof. Dr. med. Harald Klüter**
Geschäftsstelle: Dr. sc. hum. Stefan Gorbey
S. Cao, M. Goerner, K. Heberlein

Haus 42 – Ebene 3
Theodor-Kutzer-Ufer 1-3
68167 Mannheim

Telefon: +49 621 383 - 71770 / - 71776 / - 71777
Telefax: +49 621 383 - 71772
ethikkommission-II@medma.uni-heidelberg.de
www.umm.uni-heidelberg.de/forschung/medizinische-ethikkommission-ii

Mannheim, 16.10.2020

Number: **2019-1127N**

Study title: Peritoneal Flap for Lymphocele Prophylaxis Following Robotic-Assisted Laparoscopic Radical Prostatectomy with Pelvic Lymph Node Dissection
Principal Investigator: Prof. Dr. med. Philipp Nuhn
Study Centre: Urologische Klinik, Universitätsmedizin Mannheim, Theodor-Kutzer-Ufer 1-3, 68167 Mannheim

Dear Prof. Nuhn,

The Ethical Committee reviewed and approved the following human research study on 19.2.2019:

Peritoneal Flap for Lymphocele Prophylaxis Following Robotic-Assisted Laparoscopic Radical Prostatectomy with Pelvic Lymph Node Dissection

German title: **PE**ritonealflap zur Prophylaxe von **LY**mphozlen nach laparoskopischer, robotis**C**h-**A**ssistierter radikaler Prostatektomie mit pelviner Lymphade**N**ektomie = PELYCAN

Submitted documents:

- Application for ethical approval (30.01.2019),
- Patient information and written consent form (version 1.0, 30.01.2019
- Research/Study protocol (30.01.2019)

Sincerely

Medizinische Ethik-Kommission II
Medizinische Fakultät Mannheim
der Universität Heidelberg
Haus 42, Ebene 3
Theodor-Kutzer-Ufer 1-3
68167 Mannheim
Tel.: +49 (0) 621/383-9706, Fax: -9710
ethikkommission-II@medma.uni-heidelberg.de
www.uni-heidelberg.de/inst/ethikkommission

Signature of chairman i. A. Name: Harald Klüter, M.D. Date: 16.10.2020

Universitätsklinikum Mannheim GmbH
Theodor-Kutzer-Ufer 1–3, 68167 Mannheim
Registergericht: Amtsgericht Mannheim, HRB Mannheim 7331
Aufsichtsratsvorsitzender: Oberbürgermeister Dr. Peter Kurz
Geschäftsführer: Freddy Bergmann, Prof. Dr. med. Hans-Jürgen Hennes

Medizinische Fakultät Mannheim
der Universität Heidelberg
Theodor-Kutzer-Ufer 1-3, 68167 Mannheim
Dekan: Prof. Dr. med. Sergij Goerdt
